# Is LRRK2 the missing link between inflammatory bowel disease and Parkinson’s disease?

**DOI:** 10.1038/s41531-021-00170-1

**Published:** 2021-03-09

**Authors:** Mary K. Herrick, Malú G. Tansey

**Affiliations:** Department of Neuroscience and Center for Translational Research in Neurodegenerative Disease at The University of Florida College of Medicine, Gainesville, FL USA

**Keywords:** Parkinson's disease, Chronic inflammation, Mutation

## Abstract

Links that implicate the gastrointestinal system in Parkinson’s disease (PD) pathogenesis and progression have become increasingly common. PD shares several similarities with Crohn’s disease (CD). Intestinal inflammation is common in both PD and CD and is hypothesized to contribute to PD neuropathology. Mutations in leucine-rich repeat kinase 2 (LRRK2) are one of the greatest genetic contributors to PD. Variants in LRRK2 have also been associated with increased incidence of CD. Since its discovery, LRRK2 has been studied intensely in neurons, despite multiple lines of evidence showing that LRRK2 is highly expressed in immune cells. Based on the fact that higher levels of LRRK2 are detectable in inflamed colonic tissue from CD patients and in peripheral immune cells from sporadic PD patients relative to matched controls, we posit that LRRK2 regulates inflammatory processes. Therefore, LRRK2 may sit at a crossroads whereby gut inflammation and higher LRRK2 levels in CD may be a biomarker of increased risk for sporadic PD and/or may represent a tractable therapeutic target in inflammatory diseases that increase risk for PD. Here we will focus on reviewing how PD and CD share overlapping phenotypes, particularly in terms of LRRK2 in the context of the immune system, that could be targeted in future therapies.

## Introduction

The *Leucine-rich repeat kinase 2* (*LRRK2)* gene has an important role in both sporadic and familial Parkinson’s disease (PD). Dominantly inherited monogenic mutations in the *LRRK2* gene, in particular at c.6055 G > A that results in an amino acid substitution in the LRRK2 protein creating the LRRK2 p.G2019S heterozygote status, confer the highest genotypic and population attributable risk for PD, but the frequency of these mutations is low in the general population. However, other *LRRK2* genetic variants, such as A419V, R1628P, S1647T, M1646T, G2385R, and N2081D, are associated with low risk for PD and are far more common in the general population^1^. Interestingly, several genome-wide association studies (GWAS) have identified that the *LRRK2* gene is a common susceptibility locus for both PD and Crohn’s disease (CD), one of the inflammatory bowel diseases (IBD) that causes chronic inflammation of the gastrointestinal (GI) tract^2–7^. On initial observation, it may seem strange that LRRK2 is linked to an inflammatory disease of the gut when LRRK2 has been heavily studied in the context of PD; however, these two seemingly unrelated diseases are more related than we realize.

Over the past decade the role of the GI system in PD has received considerable attention. It is becoming increasingly evident that there are several links between PD pathophysiology and dysfunction in the GI system, a hypothesis first proposed by Braak et al.^8^ based on α-synuclein (α-syn) immunostaining which he used to stage PD pathology from the periphery to the brain. The potential role of the gut in PD pathogenesis and pathophysiology has been thoroughly reviewed^9–11^, and overwhelming evidence has led to the hypothesis that intestinal inflammation induced by multiple types of GI perturbations may promote peripheral inflammation that could contribute to neuroinflammation and neuropathology associated with PD. While CD pathology has no known connection to the brain, CD arises with a leaky gut and subsequent aberrant immune responses and gut dysbiosis^12,13^. Given the implication of LRRK2 in CD, the PD field can look at CD to further understand LRRK2’s role in the immune system that may translate to PD or serve as a potential biomarker prior to a PD diagnosis. Here, we will review the evidence suggesting links between PD and CD and how the role of LRRK2 specifically in the immune system may contribute to PD and CD and may provide rationale to target LRRK2 in inflammatory diseases.

### Epidemiological evidence between PD and CD

Several epidemiological studies have linked PD and IBD. Some of the first studies from Taiwanese groups identified that patients with IBD have a 35% increased risk of PD^14,15^. Subsequent studies in the US^16^, Korea^17^, and Sweden^18^ identified that patients with either CD or ulcerative colitis (UC) had increased risk for PD relative to healthy controls; however, a study from Denmark only found an association between UC and PD^19^. Conversely, another study examining this association in Medicare beneficiaries found the opposite association whereby a diagnosis of IBD was associated with lower incidence of PD^20^. These conflicting results could potentially be due to chronic anti-inflammatory regimens, highlighting how differences between populations and reporting and analysis methods are important determinants of how the associations are determined and interpreted. Given these seemingly disparate findings, a systematic review and meta-analysis looking at all studies to date found overall that IBD patients had a 46% increased risk of PD (risk ratio 1.41, 95% confidence interval 1.19–1.66), and this increased risk remained when examining patients with CD (28%, risk ratio 1.28, 95% confidence interval 1.08–1.52) and UC (30%, risk ratio 1.30, 95% confidence interval 1.15–1.47) separately^21^. Interestingly, two of the studies that found a positive association between IBD and risk for PD also found that currently available anti-TNF therapy reduced the risk of developing PD^16,17^. Furthermore, these studies have raised the interesting possibility that novel soluble TNF-selective non-immunosuppressive anti-TNF therapies may afford potential therapeutic benefit to reduce risk for PD or slow its progression.

It is important to note that none of these associations should be taken as proof of a causal association since more rigorous case–control studies are needed to confirm or reject these associations in a robust cause and effect manner. However, these associations can serve as the basis for more mechanism-based studies in animal models that can interrogate cause and effect between two conditions. Additional research is needed to identify best therapeutic windows for these and other immunomodulatory drugs, and importantly to determine whether they should be given prophylactically to reduce risk for PD later in life or shortly after the onset of non-motor symptoms to delay or prevent progression of disease and the onset of the disabling motor symptoms typically associated with clinical stages of the disease.

### GI dysbiosis in PD and CD

Changes in gut microbiota composition have also been linked to increased risk for PD. The relative abundance of certain families of bacteria are notably different between PD patients and healthy controls. While several studies have noted different patterns of changes, fecal levels of *Bifidobacteriaceae*, *Bacteroides*, *Prevotellaceae*, *Christensenellaceae*, *Tissierellaceae*, *Lachnospiraceae*, *Enterobacteriaceae*, *Lactobacillaceae*, *Pasteurellaceae*, and *Verrucomicrobiaceae*, among others, have all been shown to be significantly altered in PD patients relative to healthy controls^22–24^. Some of these changes were associated with motor and non-motor symptoms. The fecal abundance of *Enterobacteriaceae* was positively associated with gait difficulty and postural instability, while decreased levels of *Lachnospiraceae* correlated with motor impairment and disease severity^23,25^. Patients with tremors exhibited relatively higher abundances of fecal *Bacteroides* relative to patients without tremors^26^. Classifying PD patients based on GI non-motor symptoms, the abundance of *Prevotellaceae* was found to be reduced in constipated patients relative to non-constipated patients, while diversity of *Firmicutes* was increased in constipated patients^27^. Levels of the anti-inflammatory bacteria from the genera *Blautia*, *Coprococcus*, and *Roseburia* were reduced in the colons of PD patients relative to healthy controls, while levels of the pro-inflammatory bacteria of the genus *Ralstonia* were elevated in PD patients^28^. In accordance with these bacterial changes promoting an inflammatory environment in the GI system of PD patients, levels of immune factors, such as Flt1, IL-1α, and CXCL8 have been shown to be elevated in PD patient stool^29^, while levels of *Bacteroides* and *Verrucomicrobia* have been positively correlated with plasma levels of TNF and interferon gamma (IFNγ), respectively^26^. Thus, changes in microbiome could potentially be used as biomarkers for disease state or risk. For more detailed analyses reviewing GI dysbiosis in PD, we refer the readers to ref. ^30–33^.

In a similar fashion, GI dysbiosis has been associated with IBD. Fecal samples from CD patients exhibit higher levels of *Enterobacteriaceae*, *Fusobacterium*, and *Enterococcus faecalis*^34–36^. Levels of fecal *Escherichia coli* positively correlate, while fecal levels of *Bacteroides* negatively correlate with age of CD patients^37^. On the contrary, CD patients have been noted with an overall decrease in microbial diversity due to lower levels of *Firmicutes* with 43 ribotypes identified in healthy patients compared to only 13 in CD patients^38^. In the colon, *Lachnospiraceae*, *Firmicutes*, and *Bacteriodetes* have all been shown to be reduced in the mucosa in CD patients relative to healthy controls^39,40^. Previous studies have identified adherent-invasive *Escherichia coli* in active phases of CD and *Mycobacterium avium paratuberculosis* as potential causal roles of CD, suggesting a link between CD patients and opportunistic pathogens that prime the environment for ensuing alterations in intestinal permeability and inflammation^41,42^.

Despite these reports noting several differences in the gut microbiota composition of PD or CD patients relative to healthy controls, these findings are not conclusive and need to be reproduced to confirm correlations, which is often difficult in the microbiome field given the number of different factors that can disrupt the microbiome and the lack of standardization of experimental and analytical methods. Furthermore, it is extremely important to note that the implications of microbiome changes documented in PD are not well understood. It is unknown whether microbiome changes alter GI dysfunction, which in turn contributes to prodromal PD, or vice versa. The mechanisms by which these changes occur or how to effectively target these bacteria as a potential therapeutic to delay or mitigate the pathogenesis of PD or CD still remain unknown. However, these data support the hypothesis that gut dysbiosis could be one of many peripheral perturbations that could promote GI inflammation, which may be one of the key initiating events in PD pathogenesis^9^.

### Intestinal and peripheral inflammatory phenotypes in PD and CD

**Fig. 1:**
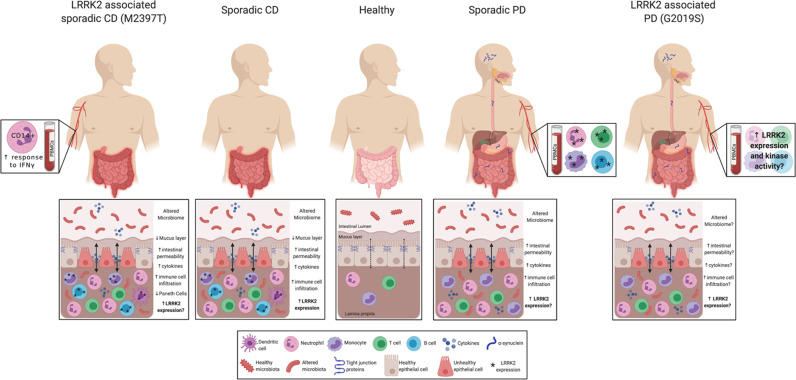
LRRK2 immune cell expression at the interface of PD and CD. PD and CD exhibit very similar intestinal phenotypes with altered microbiome, increased intestinal permeability, cytokine secretion, and immune cell infiltration. While LRRK2 expression is increased in specific immune cells in the inflamed intestine in sporadic CD, LRRK2 expression in intestinal immune cells from PD patients has not been assessed. However, LRRK2 expression is increased in sporadic PD patient PBMCs, and CD14+ monocytes from LRRK2 M2397T CD patients have increased responses to IFNγ. While little is known about the LRRK2 expression and activity in the GI and peripheral immune systems of LRRK2 G2019S PD patients, we hypothesize that they will present with higher levels of LRRK2 expression and kinase activity and similar GI phenotypes as patients with non-LRRK2 PD. Given the current evidence for LRRK2’s role in sporadic and genetic PD and CD, we posit that LRRK2 regulates inflammatory responses in the periphery and this is the reason its levels are increased in chronic inflammatory conditions which may serve as a potential therapeutic. Figure created with Biorender.com.

Beyond association studies and GI dysbiosis, PD and IBD patients exhibit similar intestinal and peripheral phenotypes especially in the context of inflammation and gut permeability that are summarized in Fig. 1. PD and CD patients exhibit increased intestinal permeability potentially due to disruptions and decreased expression of tight junction proteins^12,13,43,44^. In CD, tight junction proteins present distinct patterns of distribution with claudin-2 highly upregulated in inflamed epithelium, and claudin-3 and -4 downregulated and redistributed in diseased epithelial cells^45^. In parallel, claudin-5 and -8 are downregulated and localized away from the tight junction^13^, while occludin and zona occludens-1 (ZO-1) are significantly reduced in CD patients^46,47^. IFNγ contributes to decreased ZO-1 and occludin by causing increased internalization of the proteins away from the epithelium membrane in CD^47^. In PD, studies have shown similar phenotypes with a reduction of occludin and redistribution of both occludin and ZO-1 in colonic biopsies from PD patients relative to healthy controls^48^; however, further studies are needed to fully assess the epithelial barrier of PD patients. We would predict that the severity of disruption in the intestinal tight junction proteins may be correlated with disease pathology which may serve as a potential biomarker for PD prior to the onset of disabling motor PD symptoms.

Disruption of gut barrier integrity and increased intestinal permeability is hypothesized to promote intestinal inflammation and systemic peripheral inflammation, phenotypes in which PD and CD share similarities. Pro-inflammatory cytokines, including IL-1β, TNF, IFNγ, IL-2, IL-6, and CXCL8, are associated with initiation and progression of IBD^49^, and many of these are the same cytokines found to be increased in the sera, cerebrospinal fluid (CSF), and brain of PD patients relative to age-matched healthy controls^50–55^. One of the major cytokines increased in PD, TNF, is considered a major contributor to IBD pathogenesis through its pleotropic effects in a number of signaling cascades^56^. Similar to IBD patients, PD patients exhibited increased mRNA levels of pro-inflammatory cytokines in the ascending colon relative to healthy controls^57^. Interestingly, cytokine levels negatively correlated with disease duration, suggesting intestinal inflammation may be a precursor or contributes to initiation of PD pathogenesis but does not remain at high levels throughout the progression of disease^57^. Alternatively, cells that produce these cytokines may be transient and become less frequent through disease progression; thus, further assessment of the mechanism behind this correlation is needed. Prospective studies in asymptomatic individuals at risk for PD are needed to identify the causes of these relationships to understand how these inflammatory abnormalities in cytokine levels influence PD pathogenesis.

### LRRK2 variants implicated in PD and CD

In 2002, the PARK8 locus on chromosome 12 was identified in a Japanese family with autosomal dominant PD^58^. In 2004, missense autosomal dominant mutations in *LRRK2* were identified as a causative form of dominantly inherited PD^59,60^. In 2008 a GWAS implicated LRRK2 in CD, and this was further confirmed in a subsequent study in populations with European descent^5,7^.

LRRK2 is a large protein containing two functional domains—a Ras of complex (ROC) with intrinsic GTPase domain and a serine/threonine kinase domain. Many of the pathogenic PD mutations reside in these functional domains, including N1437H, R1441G/C/H, and Y1699C in the ROC (Ras of complex proteins) and COR (C-terminal of Roc) domains and I2012T, G2019S, and I2020T in the kinase domain. These mutations generally result in decreased GTPase activity or increased kinase activity, respectively. Aside from these pathogenic PD mutations, the M2397T variant in the WD40 domain has been associated with sporadic CD and shown to affect LRRK2 protein levels^7,61,62^. The newly identified N2081D variant is associated with increased risk for both CD and PD while the N551K and R1398H variants are associated with reduced risk for both diseases^2,63^. Interestingly, the N2081D variant falls in the same kinase domain as the p.G2019S mutation and results in increased kinase activity, albeit not to the same level as the p.G2019S mutation, while the R1398H variant that falls in the ROC domain has been shown to deactivate LRRK2 by increasing GTPase activity^2^. This suggests that PD and CD pathogenesis may be closely linked to specific LRRK2 functions directly related to the GTPase and kinase domains.

### LRRK2 in PD and CD immune cells

Since the identification of pathogenic LRRK2 mutations in PD, most research has focused on the role of LRRK2 in neurons as LRRK2 gain-of-function kinase activity has been shown to affect neurite outgrowth^64,65^. However, recent advances into the expression and function of LRRK2 in the context of the immune system have garnered considerable attention.

LRRK2 is most highly expressed in peripheral blood mononuclear cells (PBMCs). In fact, LRRK2 expression in leukocytes is much higher than that in neurons^66,67^. CD14+ monocytes, neutrophils, CD19+ B cells, CD4+ T cells, and CD8+ T cells from healthy control PBMCs all express LRRK2; however, expression is highest in CD14+, CD16+ monocytes, and neutrophils^68–71^. Stimulation with lipopolysaccharide (LPS) or IFNγ upregulates LRRK2 expression in myeloid cells from primary monocytes^68,72^, bone-marrow derived macrophages^68^, or human THP-1 monocytic leukemia cell lines^73^. While LRRK2 expression is relatively low in T cells compared to other immune cell types in peripheral blood, its expression can still be induced by IFNγ stimulation^70,72^. In PBMCs from sporadic PD patients, LRRK2 expression in B cells, T cells, and CD16+ monocytes is higher relative to healthy controls, and the relationship between monocyte LRRK2 levels and inflammatory activity is significantly different in PD patients as compared to healthy controls^74^. While the functional significance of these findings has not been elucidated, these findings imply that LRRK2 increases either drive or respond to inflammation in peripheral blood.

Similar to PD, LRRK2 is present in immune cells from CD patients, and that LRRK2 expression is upregulated with IFNγ stimulation^72^. LRRK2 mRNA is upregulated in inflamed CD intestinal tissue relative to uninflamed tissue from the same patient, and this LRRK2 expression was localized to macrophages, B cells, and dendritic cells in the lamina propria, a thin layer of connective tissue that lines the intestinal tract^72^. A recent study identified that CD14+ monocytes from the blood of CD patients with the LRRK2 M2397T variant exhibit an elevated pro-inflammatory response to IFNγ relative to healthy controls^75^. Paneth cells are specialized secretory cells in the small intestine that regulate intestinal microbiome and innate immune response. Interestingly, LRRK2 M2397T is associated with Paneth cell defects in Japanese CD patients as the number of M2397T alleles negatively correlates with number of normal Paneth cells and pathway analysis suggests this may be due to defects in autophagy^76^, one of the many pathways in which LRRK2 has been implicated.

Given LRRK2 expression has been identified in immune cells in the context of both PD and CD, it could be hypothesized that LRRK2 may play a role in the induction of α-syn within immune cells. In 2017, Stolzenberg et al.^77^ showed that α-syn expression in enteric neurites positively correlated with the degree of acute and chronic inflammation, and individuals infected with norovirus, an infection that causes gastroenteritis, showed temporary increased α-syn misfolding and aggregation that could contribute to chemoattractant migration of neutrophils and monocytes. This study supports the hypothesis that α-syn pathology could potentially begin in the gut and is influenced by peripheral inflammation. In theory, LRRK2 could contribute to this observation, given its high expression in neutrophils and monocytes; however, this has not been reported. Therefore, further analysis of gut biopsies from PD and IBD patients with a detailed and complete medical history including immune-related conditions should be assessed for LRRK2 expression. In addition, further research needs to focus on the exact mechanisms by which LRRK2 acts at the immune cell level to induce or alter α-syn pathology, especially in the context of PD.

The current understanding of LRRK2 in the context of the immune system of PD and CD LRRK2 and non-LRRK2 patients is outlined in Fig. 1. Studies suggest that LRRK2 regulation of immune cell function is cell type- and stimulus-specific and dependent on LRRK2 kinase activity, and this has been thoroughly reviewed^78,79^. The fact that LRRK2 expression is high in PBMCs under homeostatic conditions and further increased upon stimulation suggests its induction is likely to play a regulatory role in their effector functions; therefore, caution should be exercised when targeting LRRK2 as a potential therapeutic intervention in PD or CD as it is still unclear whether the high LRRK2 levels in immune cells are a protective or deleterious mechanism in the immune system. Additional translational studies are needed to fully understand the normal function of LRRK2 in the context of human immune cells, as well as the potential pathogenic role of LRRK2 mutations in immune cells, both of which should be possible with greater access to LRRK2 PD and CD patient blood samples and/or via utilization of human induced pluripotent stem cell-derived macrophages, monocytes, and microglia from LRRK2 PD and CD patients^80,81^. Our ability to investigate the cell-type specific role of LRRK2 will open up new avenues to target its function in a way that we can treat gain-of-function mutation effects in humans in the clinic.

### LRRK2 in colitis models

Although a plethora of evidence suggests a link between PD and IBD with LRRK2 function seemingly at the interface between the two diseases, very limited studies have examined LRRK2 function within the scope of both diseases. To date, few studies have been published examining colitis in LRRK2 genetic mouse models^62,82^. The gold standard for studying colitis in rodent models is the use of dextran sodium sulfate salt (DSS) or trinitrobenzenesulfonic acid (TNSB), both of which have been heavily used in the GI field due to their simplicity and reproducibility^83,84^. The exact molecular mechanisms by which these chemicals induce colitis phenotypes remain unknown, but they basically induce epithelial injury followed by inflammation. While these are particularly useful models, it is important to note they differ in phenotypic presentation order from actual IBD with rodent models subjected to DSS developing intestinal inflammation after destruction of the epithelial lining and increased intestinal permeability whereas the human IBDs result from an imbalance of the immune system in the intestine followed by ensuing microbial dysbiosis and gut alterations^85,86^. Typically included in the drinking water of rodents, DSS paradigms can vary greatly with colitogenic properties dependent on dosage, molecular weight of DSS, and administration length (acute vs. chronic). All of these factors should be considered when assessing and interpreting effects of colitis models on the gut–brain axis.

In 2011, Liu et al.^62^ induced colitis in LRRK2 knockout mice using acute DSS and reported that LRRK2 knockout mice were more susceptible to DSS-induced colitis relative to wildtype controls. The authors hypothesized that there was an exacerbated inflammatory response in the context of LRRK2 deficiency due to increased NFAT activation in macrophages^62^. In the presence of LRRK2, NFAT transport to the nucleus was blocked by LRRK2 interacting with the NRON scaffolding complex^62,87^. Based on their model, LRRK2 deficiency would be expected to result in NFAT translocation to the nucleus, thereby triggering increased IFNγ transcription^87,88^. However, a 2018 study by Takagawa et al. was not able to replicate these data but extended the findings, suggesting BAC transgenic mice overexpressing wildtype LRRK2 are more susceptible to acute DSS-induced colitis than wildtype mice. They attributed the phenotype in part to Dectin-1 stimulation in dendritic cells that leads to dysregulated inflammatory signaling through the NFκB pathway^82^. The authors proposed a model in which LRRK2 dephosphorylates Beclin-1, thereby preventing its degradation, blocking autophagy, and increasing LRRK2 expression, all of which were ameliorated with LRRK2 kinase inhibitors^82^.

While the mechanism by which LRRK2 alters inflammation in the context of colitis models is still being explored, additional studies have suggested other hypotheses related to LRRK2 expression and its kinase activity in the gut. LRRK2 phosphorylation increases in inflamed colonic mucosa concomitant with IFNγ production after acute colitis, consistent with the idea that LRRK2 expression is regulated in an IFNγ-dependent manner^89^. In addition, LRRK2 may disrupt T-helper 17 (Th17) levels and function. Present in the lamina propria of the intestine, Th17 cells are key orchestrators in mucosal homeostasis and respond to gut pathogens such that, when they are dysfunctional, Park et al.^90^ hypothesized they contribute to LRRK2-dependent intestinal inflammation. In this manner, LRRK2 G2019S may suppress Th17 activity and differentiation in the gut due to an increase in immature myeloid cells, which would be expected to be reversed with LRRK2 kinase inhibition^90^.

Studies examining LRRK2 in colitis models have been limited in scope and do not examine the effects of intestinal inflammation on PD-associated pathology in either the nigrostriatal pathway or the GI system as other studies have done in wildtype animal models detailed below. Given that intestinal inflammation is common to both PD and CD and that intestinal inflammation is hypothesized to contribute to GI pathology that precedes a PD clinical diagnosis, more in-depth studies need to be conducted to further examine the causal links between the two diseases.

### Evidence linking colitis and PD-associated neuropathology in animal models

Although DSS has been comprehensively used to study colitis in terms of the gut and immune cell alterations, increasing evidence suggests that intestinal inflammation induced by DSS can cause alterations in the brain. Some of the earliest reports showed that experimental colitis increases the permeability of the blood–brain barrier (BBB) evinced by increased leakage of sodium fluorescein in a rabbit model^91^ and 1–2 days after colitis induction in the hypothalamus of a rat model^92^. More specifically, occludin and claudin-5 were reduced in the hippocampus of mouse brains after acute colitis, further suggesting impairment of BBB integrity after colitis^93^. Beyond changes in BBB integrity, changes related to inflammatory cytokines and cytokines associated with peripheral immune cell trafficking to the central nervous system (CNS) have been shown to be altered in the brain after colitis induction. Inflammatory IL-6, a pleotropic cytokine with both pro- and anti-inflammatory properties depending on the physiological state is highly upregulated in the periphery after colitis and has also been shown to be increased in the brain after DSS- or TNBS-induced colitis, albeit in a temporal manner with increased IL-6 in the cerebral cortex and hypothalamus of rats following colitis induction^93,94^. Similarly TNF has been shown to be increased in the cortex, while COX-2 mRNA was increased in the hippocampus and hypothalamus but reduced in the amygdala, again suggesting brain region-specific spatiotemporal alterations of inflammatory markers after colitis^93,95^. IL-1β has been shown to be upregulated in the substantia nigra (SN) of mice after acute and sub-chronic DSS-induced colitis^96^. Furthermore, endothelial vascular cell adhesion molecule 1 (V-CAM1), a cytokine-inducible molecule that mediates lymphocyte adhesion, is upregulated in the brain of rats and mice after TNSB- or DSS-induced colitis, and this upregulation has been positively correlated with colonic inflammation and colonic V-CAM1 levels^97^. While V-CAM1 upregulation was not associated with leukocyte infiltration into the brain in the latter study, it could be hypothesized that it was examined too early in the process and leukocyte infiltration might have been detectable only at a later time point.

More recently, studies have examined other effects of colitis on the brain, specifically in relation to glial cells. Astrogliosis has been in observed in DSS-treated animals, as GFAP mRNA and protein levels were increased in the hippocampus^95^. Alterations in microglia phenotypes have been noted in the prefrontal cortex with a shift to a more pro-inflammatory and damage-associated microglia phenotype and this was concomitant with increased peripheral monocyte infiltration in the CNS^98^. Similarly, acute DSS increased peripheral monocyte infiltration into the hippocampus of wildtype mice in conjunction with increased peripheral and brain pro-inflammatory cytokine levels^99^.

Importantly, a few recent studies have reported that colitis is sufficient to induce alterations in the dopaminergic nigrostriatal pathway. A reduction in expression of tyrosine hydroxylase (TH), the rate limiting enzyme in dopamine synthesis, in the SN was observed in a study using acute DSS alone^100^; however, DSS in conjunction with the neurotoxin MPTP, resulted in enhanced reduction of TH expression in the SN, as well as increases in Iba1+ cells and GFAP+ cells, suggestive of a degenerative phenotype with astrogliosis and increased microglial activation^100^. In a separate study examining different paradigms of DSS induction, it has been reported that an acute model is sufficient to increase nigral IL-1β, suggesting alterations in the inflammatory state of the SN, but insufficient to produce nigrostriatal degenerative phenotypes. Similarly, a sub-chronic paradigm also induced nigral IL-1β increases; however, unlike the acute paradigm, the subchronic induction was sufficient to alter the nigrostriatal pathway with reduced TH expression in the SN and decreased striatal dopamine, suggestive of a neurodegenerative phenotype^96^. A study examining a double-hit model with LPS injected in the SN of rats in conjunction with acute colitis resulted in exacerbated BBB permeability, peripheral and neuroinflammation, and dopaminergic neuronal loss; and these features were ameliorated with depletion of peripheral macrophages, promoting the idea that brain neuropathology and neuroinflammation are modulated by peripheral inflammation^101^.

Collectively, these data strengthen the functional links between the gut–brain axis and peripheral circulation and provide evidence that intestinal inflammation or GI perturbations may promote PD pathogenesis and/or disease progression. While Braak’s hypothesis might suggest the direction of the communication is solely gut-to-brain, data from several studies suggest that the communication could be bidirectional^96,102,103^.

## Conclusions

An overwhelming amount of data now support that the anatomical link between the GI system and the brain may play a role in the pathophysiology of PD; yet the extent to which GI dysfunction plays a role in pathogenesis of PD needs to be defined more clearly so therapies to prevent or arrest progression of disease can be developed and moved into the clinic. Specifically, understanding the similarities in phenotypes between PD and peripheral inflammatory diseases of the gut previously not believed to be risk factors for PD may provide insight into PD pathogenesis and underlying disease mechanisms for potential therapeutic intervention that could mitigate risk for multiple inflammatory diseases that increase risk for age-related diseases that are associated with neuroinflammation like PD. Mechanistically, given that LRRK2 sits at the interface between PD and CD and that studies have shown increased LRRK2 levels in peripheral immune cells of PD patients^74^ or in inflamed colonic tissue of CD patients^72^, future studies should directly investigate the role that LRRK2 plays in the gut–brain axis in PD and how LRRK2 synergizes with intestinal inflammation to promote neuroinflammation and neuropathology associated with PD.

## References

[1] Christensen, K. V. et al. LRRK2 exonic variants associated with Parkinson’s disease augment phosphorylation levels for LRRK2-Ser1292 and Rab10-Thr73. *bioRxiv*10.1101/447946 (2018).

[2] Hui, K. Y. et al. Functional variants in the LRRK2 gene confer shared effects on risk for Crohn’s disease and Parkinson’s disease. *Sci. Transl. Med.*10.1126/scitranslmed.aai7795 (2018). 10.1126/scitranslmed.aai7795 PMC6028002 29321258

[3] Michail S, Bultron G, Depaolo RW (2013). Genetic variants associated with Crohn’s disease. Appl. Clin. Genet..

[4] Umeno J (2011). Meta-analysis of published studies identified eight additional common susceptibility loci for Crohn’s disease and ulcerative colitis. Inflamm. Bowel Dis..

[5] Franke A (2010). Genome-wide meta-analysis increases to 71 the number of confirmed Crohn’s disease susceptibility loci. Nat. Genet..

[6] Hugot JP (2001). Association of NOD2 leucine-rich repeat variants with susceptibility to Crohn’s disease. Nature.

[7] Barrett JC (2008). Genome-wide association defines more than 30 distinct susceptibility loci for Crohn’s disease. Nat. Genet..

[8] Braak H (2003). Staging of brain pathology related to sporadic Parkinson’s disease. Neurobiol. Aging.

[9] Houser MC, Tansey MG (2017). The gut-brain axis: is intestinal inflammation a silent driver of Parkinson’s disease pathogenesis?. NPJ Parkinsons Dis..

[10] Scheperjans F, Derkinderen P, Borghammer P (2018). The gut and Parkinson’s disease: hype or hope?. J. Parkinsons Dis..

[11] Liddle RA (2018). Parkinson’s disease from the gut. Brain Res..

[12] Michielan A, D’Inca R (2015). Intestinal permeability in inflammatory bowel disease: pathogenesis, clinical evaluation, and therapy of leaky gut. Mediat. Inflamm..

[13] Zeissig S (2007). Changes in expression and distribution of claudin 2, 5 and 8 lead to discontinuous tight junctions and barrier dysfunction in active Crohn’s disease. Gut.

[14] Lin JC, Lin CS, Hsu CW, Lin CL, Kao CH (2016). Association between Parkinson’s disease and inflammatory bowel disease: a Nationwide Taiwanese Retrospective Cohort Study. Inflamm. Bowel Dis..

[15] Lai SW, Liao KF, Lin CL, Sung FC (2014). Irritable bowel syndrome correlates with increased risk of Parkinson’s disease in Taiwan. Eur. J. Epidemiol..

[16] Peter I (2018). Anti-tumor necrosis factor therapy and incidence of Parkinson disease among patients with inflammatory bowel disease. JAMA Neurol..

[17] Park, S. et al. Patients with inflammatory bowel disease are at an increased risk of Parkinson’s disease: a South Korean Nationwide population-based study. *J. Clin. Med.*10.3390/jcm8081191 (2019). 10.3390/jcm8081191 PMC6723604 31398905

[18] Weimers P (2019). Inflammatory bowel disease and Parkinson’s disease: a Nationwide Swedish Cohort Study. Inflamm. Bowel Dis..

[19] Villumsen M, Aznar S, Pakkenberg B, Jess T, Brudek T (2019). Inflammatory bowel disease increases the risk of Parkinson’s disease: a Danish nationwide cohort study 1977-2014. Gut.

[20] Camacho-Soto A, Searles Nielsen S, Racette BA (2018). Inflammatory bowel disease and risk of Parkinson’s disease in medicare beneficiaries. Parkinsonism Relat. Disord..

[21] Zhu F (2019). The risk of Parkinson’s disease in inflammatory bowel disease: a systematic review and meta-analysis. Dig. Liver Dis..

[22] Hasegawa S (2015). Intestinal dysbiosis and lowered serum lipopolysaccharide-binding protein in Parkinson’s disease. PLoS ONE.

[23] Pietrucci D (2019). Dysbiosis of gut microbiota in a selected population of Parkinson’s patients. Parkinsonism Relat. Disord..

[24] Hill-Burns EM (2017). Parkinson’s disease and Parkinson’s disease medications have distinct signatures of the gut microbiome. Mov. Disord..

[25] Scheperjans F (2015). Gut microbiota are related to Parkinson’s disease and clinical phenotype. Mov. Disord..

[26] Lin CH (2019). Altered gut microbiota and inflammatory cytokine responses in patients with Parkinson’s disease. J. Neuroinflamm..

[27] Zhu L (2014). Structural changes in the gut microbiome of constipated patients. Physiol. Genomics.

[28] Keshavarzian A (2015). Colonic bacterial composition in Parkinson’s disease. Mov. Disord..

[29] Houser MC (2018). Stool immune profiles evince gastrointestinal inflammation in Parkinson’s disease. Mov. Disord..

[30] Cryan JF, O’Riordan KJ, Sandhu K, Peterson V, Dinan TG (2020). The gut microbiome in neurological disorders. Lancet Neurol..

[31] Haikal C, Chen QQ, Li JY (2019). Microbiome changes: an indicator of Parkinson’s disease?. Transl. Neurodegener..

[32] Lubomski M (2020). Parkinson’s disease and the gastrointestinal microbiome. J. Neurol..

[33] Needham, B. D., Kaddurah-Daouk, R. & Mazmanian, S. K. Gut microbial molecules in behavioural and neurodegenerative conditions. *Nat. Rev. Neurosci*. 10.1038/s41583-020-00381-0 (2020). 10.1038/s41583-020-00381-0 33067567

[34] Seksik P (2003). Alterations of the dominant faecal bacterial groups in patients with Crohn’s disease of the colon. Gut.

[35] Chen L (2014). Characteristics of fecal and mucosa-associated microbiota in Chinese patients with inflammatory bowel disease. Medicines.

[36] Zhou Y (2016). Increased *Enterococcus faecalis* infection is associated with clinically active Crohn disease. Medicines.

[37] Nwosu FC (2013). Age-dependent fecal bacterial correlation to inflammatory bowel disease for newly diagnosed untreated children. Gastroenterol. Res. Pract..

[38] Manichanh C (2006). Reduced diversity of faecal microbiota in Crohn’s disease revealed by a metagenomic approach. Gut.

[39] Frank DN (2007). Molecular-phylogenetic characterization of microbial community imbalances in human inflammatory bowel diseases. Proc. Natl Acad. Sci. USA.

[40] Fujimoto T (2013). Decreased abundance of *Faecalibacterium prausnitzii* in the gut microbiota of Crohn’s disease. J. Gastroenterol. Hepatol..

[41] Li J, Butcher J, Mack D, Stintzi A (2015). Functional impacts of the intestinal microbiome in the pathogenesis of inflammatory bowel disease. Inflamm. Bowel Dis..

[42] DeGruttola AK, Low D, Mizoguchi A, Mizoguchi E (2016). Current understanding of dysbiosis in disease in human and animal models. Inflamm. Bowel Dis..

[43] Forsyth CB (2011). Increased intestinal permeability correlates with sigmoid mucosa alpha-synuclein staining and endotoxin exposure markers in early Parkinson’s disease. PLoS ONE.

[44] Salat-Foix D, Tran K, Ranawaya R, Meddings J, Suchowersky O (2012). Increased intestinal permeability and Parkinson disease patients: chicken or egg?. Can. J. Neurol. Sci..

[45] Prasad S (2005). Inflammatory processes have differential effects on claudins 2, 3 and 4 in colonic epithelial cells. Lab. Investig..

[46] Kucharzik T, Walsh SV, Chen J, Parkos CA, Nusrat A (2001). Neutrophil transmigration in inflammatory bowel disease is associated with differential expression of epithelial intercellular junction proteins. Am. J. Pathol..

[47] Scharl M, Paul G, Barrett KE, McCole DF (2009). AMP-activated protein kinase mediates the interferon-gamma-induced decrease in intestinal epithelial barrier function. J. Biol. Chem..

[48] Clairembault T (2015). Structural alterations of the intestinal epithelial barrier in Parkinson’s disease. Acta Neuropathol. Commun..

[49] Muzes G, Molnar B, Tulassay Z, Sipos F (2012). Changes of the cytokine profile in inflammatory bowel diseases. World J. Gastroenterol..

[50] Mogi M (1994). Interleukin-1 beta, interleukin-6, epidermal growth factor and transforming growth factor-alpha are elevated in the brain from parkinsonian patients. Neurosci. Lett..

[51] Mogi M (1994). Tumor necrosis factor-alpha (TNF-alpha) increases both in the brain and in the cerebrospinal fluid from parkinsonian patients. Neurosci. Lett..

[52] Imamura K (2003). Distribution of major histocompatibility complex class II-positive microglia and cytokine profile of Parkinson’s disease brains. Acta Neuropathol..

[53] Nagatsu, T., Mogi, M., Ichinose, H. & Togari, A. Cytokines in Parkinson’s disease. *J. Neural Transm. Suppl.*, 143–151 (2000). 11128604

[54] Blum-Degen D (1995). Interleukin-1 beta and interleukin-6 are elevated in the cerebrospinal fluid of Alzheimer’s and de novo Parkinson’s disease patients. Neurosci. Lett..

[55] Eidson LN (2017). Candidate inflammatory biomarkers display unique relationships with alpha-synuclein and correlate with measures of disease severity in subjects with Parkinson’s disease. J. Neuroinflamm..

[56] Murch SH, Braegger CP, Walker-Smith JA, MacDonald TT (1993). Location of tumour necrosis factor alpha by immunohistochemistry in chronic inflammatory bowel disease. Gut.

[57] Devos D (2013). Colonic inflammation in Parkinson’s disease. Neurobiol. Dis..

[58] Funayama M (2002). A new locus for Parkinson’s disease (PARK8) maps to chromosome 12p11.2-q13.1. Ann. Neurol..

[59] Zimprich A (2004). Mutations in LRRK2 cause autosomal-dominant parkinsonism with pleomorphic pathology. Neuron.

[60] Paisan-Ruiz C (2004). Cloning of the gene containing mutations that cause PARK8-linked Parkinson’s disease. Neuron.

[61] Fava VM (2016). A missense LRRK2 variant is a risk factor for excessive inflammatory responses in leprosy. PLoS Negl. Trop. Dis..

[62] Liu Z (2011). The kinase LRRK2 is a regulator of the transcription factor NFAT that modulates the severity of inflammatory bowel disease. Nat. Immunol..

[63] Gopalai AA (2019). LRRK2 N551K and R1398H variants are protective in Malays and Chinese in Malaysia: a case-control association study for Parkinson’s disease. Mol. Genet. Genom. Med..

[64] Plowey ED, Cherra SJ, Liu YJ, Chu CT (2008). Role of autophagy in G2019S-LRRK2-associated neurite shortening in differentiated SH-SY5Y cells. J. Neurochem..

[65] Ramonet D (2011). Dopaminergic neuronal loss, reduced neurite complexity and autophagic abnormalities in transgenic mice expressing G2019S mutant LRRK2. PLoS ONE.

[66] West AB (2017). Achieving neuroprotection with LRRK2 kinase inhibitors in Parkinson disease. Exp. Neurol..

[67] Kozina E (2018). Mutant LRRK2 mediates peripheral and central immune responses leading to neurodegeneration in vivo. Brain.

[68] Hakimi M (2011). Parkinson’s disease-linked LRRK2 is expressed in circulating and tissue immune cells and upregulated following recognition of microbial structures. J. Neural Transm..

[69] Fan Y (2018). Interrogating Parkinson’s disease LRRK2 kinase pathway activity by assessing Rab10 phosphorylation in human neutrophils. Biochem. J..

[70] Thevenet J, Pescini Gobert R, Hooft van Huijsduijnen R, Wiessner C, Sagot YJ (2011). Regulation of LRRK2 expression points to a functional role in human monocyte maturation. PLoS ONE.

[71] Atashrazm F (2019). LRRK2-mediated Rab10 phosphorylation in immune cells from Parkinson’s disease patients. Mov. Disord..

[72] Gardet A (2010). LRRK2 is involved in the IFN-gamma response and host response to pathogens. J. Immunol..

[73] Kuss M, Adamopoulou E, Kahle PJ (2014). Interferon-gamma induces leucine-rich repeat kinase LRRK2 via extracellular signal-regulated kinase ERK5 in macrophages. J. Neurochem..

[74] Cook DA (2017). LRRK2 levels in immune cells are increased in Parkinson’s disease. NPJ Parkinsons Dis..

[75] Ikezu, T. et al. Crohn’s and Parkinson’s disease-associated LRRK2 mutations alter type II interferon responses in human CD14(+) blood monocytes ex vivo. *J. Neuroimmune Pharmacol*. 10.1007/s11481-020-09909-8 (2020). 10.1007/s11481-020-09909-8 PMC7718203 32180132

[76] Liu TC (2017). LRRK2 but not ATG16L1 is associated with Paneth cell defect in Japanese Crohn’s disease patients. JCI Insight.

[77] Stolzenberg E (2017). A role for neuronal alpha-synuclein in gastrointestinal immunity. J. Innate Immun..

[78] Ahmadi Rastegar D, Dzamko N (2020). Leucine rich repeat kinase 2 and innate immunity. Front. Neurosci..

[79] Wallings, R. L., Herrick, M. K. & Tansey, M. G. LRRK2 at the interface between peripheral and central immune function in Parkinson’s. *Front. Neurosci.*10.3389/fnins.2020.00443 (2020). 10.3389/fnins.2020.00443 PMC7253584 32508566

[80] van Wilgenburg B, Browne C, Vowles J, Cowley SA (2013). Efficient, long term production of monocyte-derived macrophages from human pluripotent stem cells under partly-defined and fully-defined conditions. PLoS ONE.

[81] Lee H, James WS, Cowley SA (2017). LRRK2 in peripheral and central nervous system innate immunity: its link to Parkinson’s disease. Biochem. Soc. Trans..

[82] Takagawa, T. et al. An increase in LRRK2 suppresses autophagy and enhances Dectin-1-induced immunity in a mouse model of colitis. *Sci. Transl. Med.*10.1126/scitranslmed.aan8162 (2018). 10.1126/scitranslmed.aan8162 PMC6636639 29875204

[83] Okayasu I (1990). A novel method in the induction of reliable experimental acute and chronic ulcerative colitis in mice. Gastroenterology.

[84] Wirtz S, Neufert C, Weigmann B, Neurath MF (2007). Chemically induced mouse models of intestinal inflammation. Nat. Protoc..

[85] Eichele DD, Kharbanda KK (2017). Dextran sodium sulfate colitis murine model: An indispensable tool for advancing our understanding of inflammatory bowel diseases pathogenesis. World J. Gastroenterol..

[86] Kiesler P, Fuss IJ, Strober W (2015). Experimental models of inflammatory bowel diseases. Cell Mol. Gastroenterol. Hepatol..

[87] Jabri B, Barreiro LB (2011). Don’t move: LRRK2 arrests NFAT in the cytoplasm. Nat. Immunol..

[88] Shen, X., Yang, H., Wu, Y., Zhang, D. & Jiang, H. Meta-analysis: association of Helicobacter pylori infection with Parkinson’s diseases. *Helicobacter*10.1111/hel.12398 (2017). 10.1111/hel.12398 28598012

[89] Rodrigues-Sousa T (2014). Deficient production of reactive oxygen species leads to severe chronic DSS-induced colitis in Ncf1/p47phox-mutant mice. PLoS ONE.

[90] Park J (2017). Parkinson disease-associated LRRK2 G2019S transgene disrupts marrow myelopoiesis and peripheral Th17 response. J. Leukoc. Biol..

[91] Hathaway CA, Appleyard CB, Percy WH, Williams JL (1999). Experimental colitis increases blood-brain barrier permeability in rabbits. Am. J. Physiol..

[92] Natah SS, Mouihate A, Pittman QJ, Sharkey KA (2005). Disruption of the blood-brain barrier during TNBS colitis. Neurogastroenterol. Motil..

[93] Han Y (2018). Cortical inflammation is increased in a DSS-induced colitis mouse model. Neurosci. Bull..

[94] Wang K (2010). Expression of interleukin 6 in brain and colon of rats with TNBS-induced colitis. World J. Gastroenterol..

[95] Do J, Woo J (2018). From gut to brain: alteration in inflammation markers in the brain of dextran sodium sulfate-induced colitis model mice. Clin. Psychopharmacol. Neurosci..

[96] Garrido-Gil P, Rodriguez-Perez AI, Dominguez-Meijide A, Guerra MJ, Labandeira-Garcia JL (2018). Bidirectional neural interaction between central dopaminergic and gut lesions in Parkinson’s disease models. Mol. Neurobiol..

[97] Sans M (2001). Brain endothelial adhesion molecule expression in experimental colitis. Microcirculation.

[98] Sroor HM (2019). Experimental colitis reduces microglial cell activation in the mouse brain without affecting microglial cell numbers. Sci. Rep..

[99] Gampierakis, I. A. et al. Hippocampal neural stem cells and microglia response to experimental inflammatory bowel disease (IBD). *Mol. Psychiatry*10.1038/s41380-020-0651-6 (2020). 10.1038/s41380-020-0651-6 31969694

[100] Gil-Martinez AL (2019). Local gastrointestinal injury exacerbates inflammation and dopaminergic cell death in Parkinsonian mice. Neurotox. Res..

[101] Villaran RF (2010). Ulcerative colitis exacerbates lipopolysaccharide-induced damage to the nigral dopaminergic system: potential risk factor in Parkinson’s disease. J. Neurochem..

[102] Arotcarena ML (2020). Bidirectional gut-to-brain and brain-to-gut propagation of synucleinopathy in non-human primates. Brain.

[103] Beach, T. G. et al. Vagus nerve and stomach synucleinopathy in Parkinson’s disease, incidental lewy body disease and normal elderly subjects: evidence against the “Body-First” hypothesis. *medRxiv*10.1101/2020.09.29.20204248 (2020). 10.3233/JPD-212733 PMC10082635 34151862

